# Supplementary material for: Circulating isomiRs May Be Superior Biomarkers Compared to Their Corresponding miRNAs: A Pilot Biomarker Study of Using isomiR-Ome to Detect Coronary Calcium-Based Cardiovascular Risk in Patients with NAFLD
Source: Int J Mol Sci. 2024 Jan 10;25(2):890. doi: 10.3390/ijms25020890 (PMC10815227; doi:10.3390/ijms25020890)
Supplement: Supplementary file 1 [file ijms-25-00890-s001.zip › Supp. Table 2.pdf]

Supplemental Table S2- Individual baseline selected biochemical results of study participants

Patient's code	Age (yeas)	Sex M/F	CCS based CV risk percentile~	Hepatic Fat %^	Selected laboratory results											
					Hematology			Biochemistry					Lipid Profile			
					Hb	WBC	Platelets	FPG	Hb A1c	ALT	AST	Alb	TC	TG	HDL	LDL
	¥55.00 (49.5-62.5)	69.23% M, 30.76% F	¥66.00 (0-88)	¥9.72 (7.4-19.4)	¥14.8 (14.1-15.5)	¥5.98 (5.21-7.45)	¥225 (206-252)	¥104 (85-114)	¥5.8 (5.3-6.5)	¥33 (23-58)	¥29 (23-39)	¥4.3 (4.1-4.5)	¥182 (153-219)	¥145 (117-176)	¥48 (41-54)	¥101 (79-154)
**7**	**63**	**M**	**99**	**5.39**	**13.2**	**2.72**	**49**	**154**	**6.7**	**24**	**31**	**4.1**	**164**	**176**	**50**	**79**
**1**	**62**	**M**	**91**	**5.42**	**15.1**	**5.98**	**252**	**93**	**5.8**	**74**	**59**	**4.4**	**143**	**180**	**41**	**66**
**13**	**52**	**M**	**90**	**9.72**	**14.2**	**8.84**	**142**	**106**	**4.6**	**46**	**27**	**4.5**	**94**	**82**	**35**	**43**
**9**	**48**	**M**	**86**	**6.38**	**14.4**	**4.73**	**225**	**85**	**5.3**	**19**	**24**	**4.5**	**219**	**90**	**47**	**154**
**3**	**51**	**M**	**78**	**12.64**	**15.9**	**5.95**	**241**	**96**	**5.8**	**55**	**41**	**4.4**	**194**	**158**	**62**	**101**
**11**	**46**	**M**	**77**	**9.05**	**15.3**	**6.28**	**206**	**81**	**5.3**	**27**	**20**	**4.3**	**233**	**145**	**49**	**155**
**12**	**55**	**M**	**66**	**8.44**	**15.4**	**3.95**	**198**	**111**	**6.5**	**16**	**18**	**4.2**	**182**	**106**	**48**	**113**
**4**	**47**	**M**	**60**	**24.72**	**16.0**	**6.22**	**217**	**104**	**5.2**	**82**	**40**	**4.9**	**254**	**196**	**54**	**161**
**8**	**79**	**F**	**46**	**18.66**	**14.1**	**7.54**	**227**	**114**	**6.2**	**11**	**25**	**4.0**	**206**	**117**	**61**	**122**
**2**	**66**	**M**	**0**	**19.48**	**15.0**	**7.99**	**314**	**81**	**5.9**	**68**	**39**	**4.3**	**164**	**172**	**41**	**88**
**5**	**51**	**F**	**0**	**34.56**	**11.2**	**5.88**	**287**	**162**	**6.8**	**23**	**21**	**3.6**	**153**	**125**	**39**	**89**
**6**	**57**	**F**	**0**	**8.42**	**11.2**	**7.91**	**303**	**222**	**9.4**	**33**	*	**4.0**	**151**	**133**	**61**	**63**
**10**	**56**	**F**	**0**	**19.38**	**14.8**	**5.21**	**219**	**80**	**5.1**	**58**	**38**	**4.6**	**252**	**210**	**39**	**171**

Abbreviations: CCS- Coronary calcium score, **CV**- cardiovascular, **M**- male, **F**- female, **Hb**- Hemoglobin (gr/dL), **WBC**- White blood cells, **FPG**- Fasting plasma glucose (mg/dL)**,** **Hb A1c**- Hemoglobin A1c (%), **ALT**- Alanine aminotransferase (U/L), **AST**- Aspartate aminotransferase (U/L), **Alb**- Albumin (g/dL), **TC**- Total cholesterol (mg/dL), **TG**- Triglycerides (mg/dL), **HDL**- high density lipoprotein (mg/dL), **LDL**- Low density lipoprotein (mg/dL).

~ CCS based CV risk percentile was calculated using the Coronary Artery Calcium Score and demographic parameters (MESA calculator- **Ref**), ^As measured by Magnetic resonance spectrometry, ¥Median ± Inter Quartile Range, *Missing data.
