# Supplementary material for: Circulating isomiRs May Be Superior Biomarkers Compared to Their Corresponding miRNAs: A Pilot Biomarker Study of Using isomiR-Ome to Detect Coronary Calcium-Based Cardiovascular Risk in Patients with NAFLD
Source: Int J Mol Sci. 2024 Jan 10;25(2):890. doi: 10.3390/ijms25020890 (PMC10815227; doi:10.3390/ijms25020890)
Supplement: Supplementary file 1 [file ijms-25-00890-s001.zip › Supplementary.pdf]

Supplementary information

Title: Circulating isomiRs May Be Superior Biomarkers Compared to Their Corresponding miRNAs: A Pilot Biomarker Study of Using isomiR-ome to Detect Coronary Calcium-Based Cardiovascular Risk in Patients with NAFLD

Authors: Nataly Makarenkov, Uri Yoel, Yulia Haim, Yair Pincu, Nikhil S. Bhandarkar, Aryeh Shalev, Ilan Shelef, Idit F. Liberty, Gal Ben-Arie, David Yardeni, Assaf Rudich, Ohad Etzion and Isana Veksler-Lublinsky

Supplemental Figures

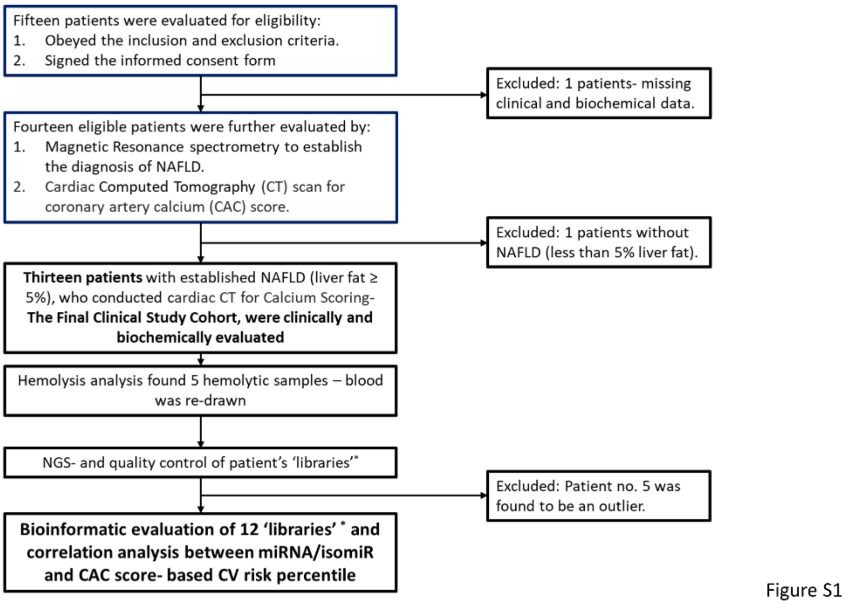

Figure S1: Patient inclusion/exclusion flow chart

Flow chart illustrating used criteria to screen the study population, including availability of clinical and biochemical data; confirmed NAFLD (liver fat content≥5%); low hemolysis level of plasma sample; NGS library passed quality controls. Fifteen patients were initially enrolled; three were excluded during the screening process; twelve patients were included in the final analyses.

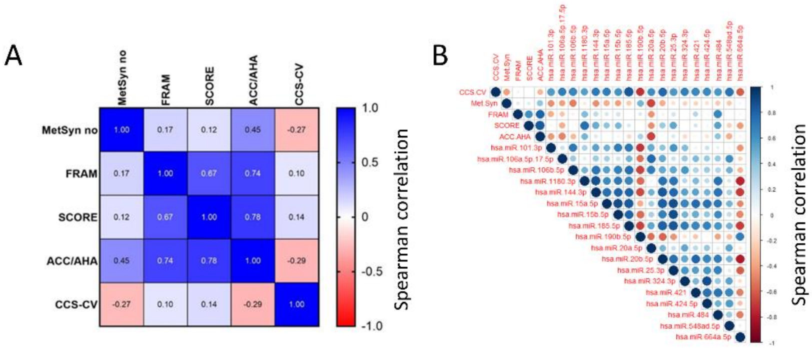

Figure S2

Figure S2: Intercorrelation between clinical scores and miRNA levels

Intercorrelations between (**A)** cardio-vascular risk calculators and (**B)** cardio-vascular risk calculators and 18 CV-risk-related miRNAs of all 12 patients in the final cohort. Each row and column represent a cardio-vascular risk calculator or a miRNA. The color and the size of each dot corresponds to correlation value. Dots are shown only for correlations with p-adj<0.1.

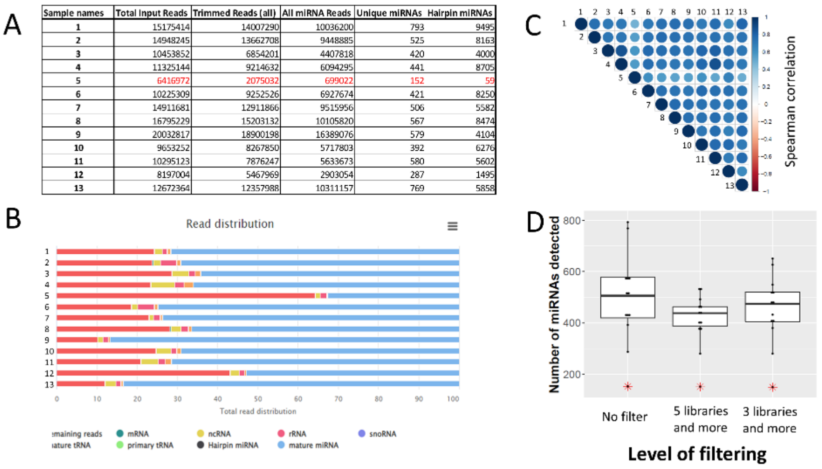

Sample names	Total Input Reads	Trimmed Reads (all)	All miRNA Reads	Unique miRNAs	Hairpin miRNAs
1	15175414	14007290	10036200	793	9495
2	14948245	13662708	9448885	525	8163
3	10453852	6854201	4407818	420	4000
4	11325144	9214632	6094295	441	8705
5	6416972	2075032	699022	152	59
6	10225309	9252526	6927674	421	8250
7	14911681	12911866	9515956	506	5582
8	16795229	15203132	10105820	567	8474
9	20032817	18900198	16389076	579	4104
10	9653252	8267850	5717803	392	6276
11	10295123	7876247	5633673	580	5602
12	8197004	5467969	2903054	287	1495
13	12672364	12357988	10311157	769	5858

Figure S3

Figure S3: Quality control of 13 libraries sequenced by NGS

(A) A summary report generated by miRGE3 for the 13 small RNA libraries that were sequenced by NGS. The library of patient 5 which was identified as an outlier is highlighted in red. (**B)** Small RNA read distribution for each sample within different types of RNA (generated by miRGE3). (**C)** Intercorrelations between libraries of all annotated miRNAs. Each row and column represents a patient. (**D).** The number of common miRNAs detected in

the libraries when looking at different levels of filtration: no filter (all unique detected miRNAs), filtered by appearance in at least 5 and at least 3 libraries. Outlier library is marked with a red asterisk (library no. 5).

Supplemental Tables

Table S1 - Individual baseline demographic and clinical characteristics of study participants

Table S2 - Individual baseline selected biochemical results of study participants

Table S3 - miRNA miEAA analysis

Table S4 - isomiR full results

Table S5 - IsomiR target pathways from DAVID
