# Supplementary material for: Circulating isomiRs May Be Superior Biomarkers Compared to Their Corresponding miRNAs: A Pilot Biomarker Study of Using isomiR-Ome to Detect Coronary Calcium-Based Cardiovascular Risk in Patients with NAFLD
Source: Int J Mol Sci. 2024 Jan 10;25(2):890. doi: 10.3390/ijms25020890 (PMC10815227; doi:10.3390/ijms25020890)
Supplement: Supplementary file 1 [file ijms-25-00890-s001.zip › Supp. Table 1.pdf]

Supplemental Table S1- Individual baseline demographic and clinical characteristics of study participants

Patient's code	Age (years)	Sex M/F	CCS based CV risk percentile ~	Hepatic Fat %^	BMI Kg/m^2	Waist circ. (Cm)	BP mmHg Sys/Dia	Medical History			Medical Treatment				
								Smoking status (pack-years)#	Pre-DM or T2DM	HTN	Aspirin*	Statins&	Anti-HTN	Anti- Diabetes	
														Metformin	Insulin§
	¥55.00 (49.5-62.5)	69.23% M, 30.76% F	¥66.00 (0-88)	¥9.72 (7.4-19.4)	¥30.78 (28.6-32.4)	¥107.00 (103.5-118.5)		23.07%	53.84%	53.84%	23.07%	46.15%	38.46%	15.38%	15.38%
7	63	M	99	5.39	27.8	107	138/83	*25*	Yes	Yes			Yes		Yes
1	62	M	91	5.42	29.4	109	118/77		Yes	Yes	Yes		Yes		
13	52	M	90	9.72	32.24	114	112/65		Yes			Yes			
9	48	M	86	6.38	30.78	141	110/74								
3	51	M	78	12.64	30.7	104	118/84					Yes			
11	46	M	77	9.05	29.61	104	132/84								
12	55	M	66	8.44	32.54	106	128/77	*30*	Yes					Yes	
4	47	M	60	24.72	26.4	94	116/79					Yes			
8	79	F	46	18.66	31.04	103	153/70			Yes	Yes		Yes		
2	66	M	0	19.48	33.8	118	146/73		Yes	Yes		Yes	Yes		
5	51	F	0	34.56	42.7	122	118/63		Yes	Yes			Yes	Yes	
6	57	F	0	8.42	31.9	119	140/95	**1**	Yes	Yes	Yes	Yes			Yes
10	56	F	0	19.38	27.5	98	125/75			Yes		Yes			

Abbreviations: CCS- Coronary calcium score, **CV**- cardiovascular, **M**- male, **F**- female, **BMI**- Body mass index, **Circ.**- Circumference, **Cm**- centimeters, **BP**- Blood pressure, **mmHg**- millimeter of mercury, **Sys**- systolic, **Dia**- diastolic, **DM**- Diabetes mellitus, **T2DM**- type 2 DM, **HTN**- Hypertension.

~ CCS based CV risk percentile was calculated using the Coronary Artery Calcium Score and demographic parameters (MESA calculator- **Ref**), ^As measured by Magnetic resonance spectrometry, #Number of years of smoking multiple cigarettes packs per day; **Bold- active smoker,** *Italian- former smoker*, *And/or other anti-Platelets drugs, &And/or ezetimibe, §Or Insulin Secretagogues, ¥Median ± Inter Quartile Range.
